# Perceptual encoding of emotions in interactive bodily expressions

**DOI:** 10.1016/j.isci.2023.108548

**Published:** 2023-11-28

**Authors:** Andrea Christensen, Nick Taubert, Elisabeth M.J. Huis in ’t Veld, Beatrice de Gelder, Martin A. Giese

**Affiliations:** 1 Section Computational Sensomotorics, Department of Cognitive Neurology, Hertie-Institute for Clinical Brain Research, Centre for Integrative Neuroscience, University Clinic Tübingen, Germany; 2 Department of Medical and Clinical Psychology, School of Social and Behavioral Sciences, Tilburg University, Tilburg, the Netherlands; 3 Brain and Emotion Laboratory, Department of Cognitive Neuroscience, Faculty of Psychology and Neuroscience, Maastricht University, Oxfordlaan 55, EV Maastricht 6229, the Netherlands

**Keywords:** Biological sciences, Behavioral neuroscience, Evolutionary biology

## Abstract

For social species, e.g., primates, the perceptual analysis of social interactions is an essential skill for survival, emerging already early during development. While real-life emotional behavior includes predominantly interactions between conspecifics, research on the perception of emotional body expressions has primarily focused on perception of single individuals. While previous studies using point-light or video stimuli of interacting people suggest an influence of social context on the perception and neural encoding of interacting bodies, it remains entirely unknown how emotions of multiple interacting agents are perceptually integrated. We studied this question using computer animation by creating scenes with two interacting avatars whose emotional style was independently controlled. While participants had to report the emotional style of a single agent, we found a systematic influence of the emotion expressed by the other, which was consistent with the social interaction context. The emotional styles of interacting individuals are thus jointly encoded.

## Highlights

• Controlled variation of emotional styles of interaction partners using computer animation
• Emotional body movements of interacting partners are jointly encoded in perception
• Perceived motion style of interaction partners modulates each other
• Perception of interaction critical for joint encoding

## Graphical abstract

**Figure:**
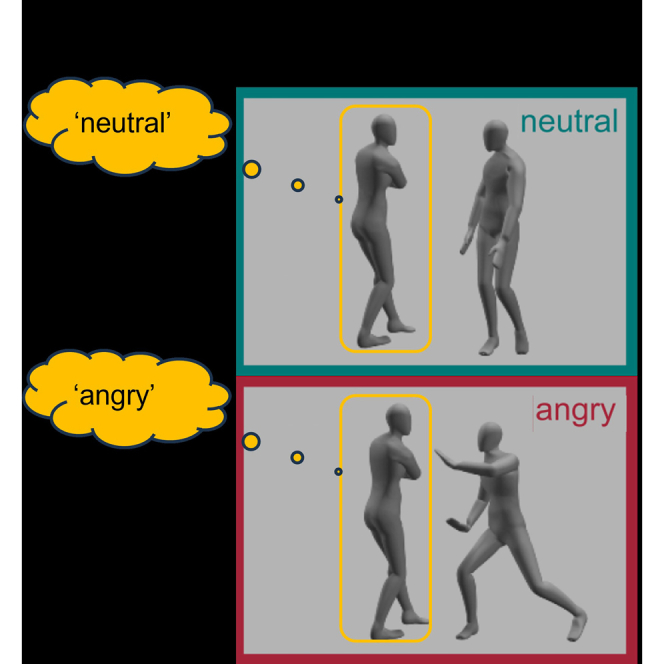

## Introduction

Only few studies have investigated the perception of emotional bodies in the presence of multiple interacting agents.^1^^,^^2^^,^^3^^,^^4^^,^^5^^,^^6^^,^^7^^,^^8^ Behavioral data show that the perception of emotional body movements is influenced by the presence of interaction partners in the scene.^7^ Functional imaging studies^6^ suggest that the neural activity induced by the perception of emotional bodies is systematically altered by the presence of other agents in the scene, and that selective emotion-specific activity can be induced by interactive stimuli, e.g., in areas like the amygdala.^9^

Since these previous experiments used point-light stimuli, or videos showing real interacting individuals, it was impossible to control their emotion content exactly. This makes it difficult to disentangle the possible influences of different cues on the perceived emotions. For example, not only were valence and arousal rated higher for interactive compared to non-interactive stimuli, but also their amount of movement.^6^

Applying techniques from computer graphics, we generated dyads of interacting agents with an exact control of the emotional style of each individual. This made it possible to study how the perception of the emotional style of one agent is influenced by the expressed emotion of the other within the context of meaningful social interactions. This technique also provides an exact control of the relevant visual form and motion features.

**Figure 1:**
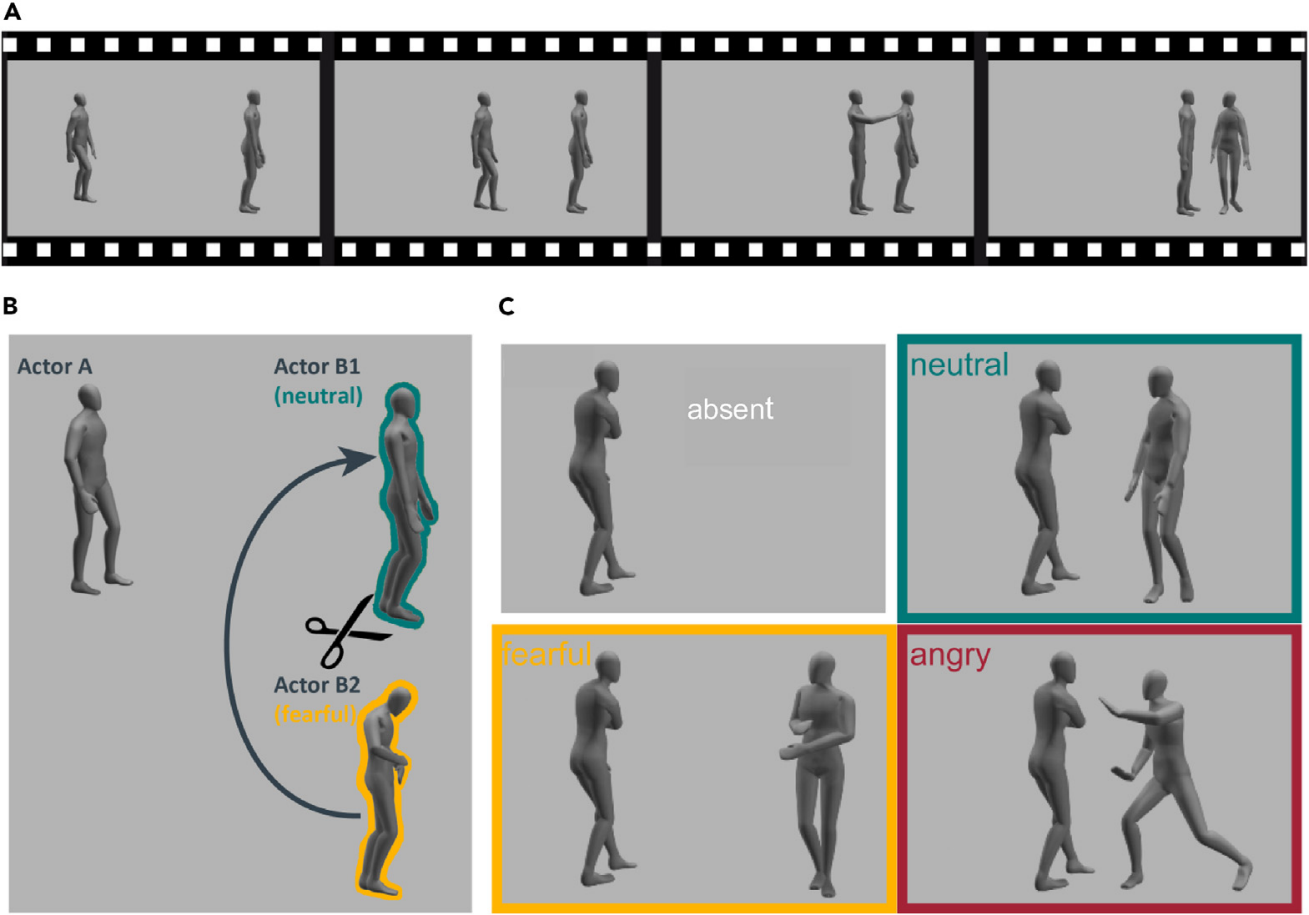
Stimuli and preparation (A) Movie showing interactive scenario: First actor (left) is approaching a second actor (right), and tapping him/her on the shoulder. The second actor turns around in different emotional styles. (B) For stimulus generation, recorded approaching and turning actions with different emotional styles were re-combined. (C) Two emotional styles of the approaching actor (angry and neural) were combined with three different types of emotional reaction (neutral, fearful, and angry), and a condition without approaching first actor (absent).

Animated stimuli were based on motion capture data recorded with a Vicon MX system using eight cameras at 120 Hz from two-person emotional interactions. Six lay actor couples executed the following social interactions: one actor approached the second and tapped him/her on the shoulder, making the second turn around and face the first (Figure 1A). The initial distance between the two actors was about 3.5 m. Both actors executed their actions either in a neutral, angry, or fearful emotional style.

To assure most emotionally expressive stimuli, we pre-selected actions and reactions for the stimulus generation. We showed real video stimuli that were recorded simultaneously with the motion capture recordings to four observers. They classified the actions and reactions of the lay actors according to prescribed response categories and rated the expressiveness of the perceived emotions, anger and fear. Only those actions and reactions that could be most reliably classified were used for stimulus generation using computer animation, exploiting the motion capture data. Since fearful approaching actions were often misclassified, we did not include those in the final experiment. Based on the ratings of the real video clips, we selected for each emotion (angry, fearful) the reaction with the highest rating. Out of the neutral reactions, we chose the one that was most reliably classified.

In a control experiment (see supplemental information), we verified that the reactions of the reacting agent were reliably classified in terms of the three selected emotion categories.

For this experiment, we did not prescribe the response categories, so that more participants were required (see the following section).

For the final experiment, we chose 12 walking and subsequent tapping actions from the approaching agent (eight angry, four neutral) and three reactions of the second agent (angry, fearful, and neutral). We created a set of standardized stimuli using commercial software (Autodesk, Motion Builder), combining each approaching and tapping action with each emotional reaction (resulting in 12 x 3 stimuli) (Figure 1B). Additionally, we created animations where the first agent was the only one present in the scene, without any induced reaction (12 stimuli) (Figure 1C). Stimuli were presented eight times in randomized order (resulting in a total of 12 × 4 × 8 = 384 stimuli), each lasting for 3–5 s.

Twenty-two subjects (five males, mean age 24.7 years) rated the anger expressed by the *approaching agent* on a Likert scale ranging from 1 “not angry” to 5 “extraordinarily angry” by pressing an appropriate key, with the explicit instruction to ignore the second agent. The number of tested subjects was well above the *a priori* estimated minimal sample sizes. Using G∗power1, these sizes were estimated for the factors Agent-Emotion and Reaction to be 10 and 13 subjects, respectively (See STAR methods for details). Responses were analyzed with a repeated measures ANOVA using the factors *Agent-Emotion* (angry, neutral) and *Reaction* (none, neutral, fearful, angry).

## Results

**Figure 2:**
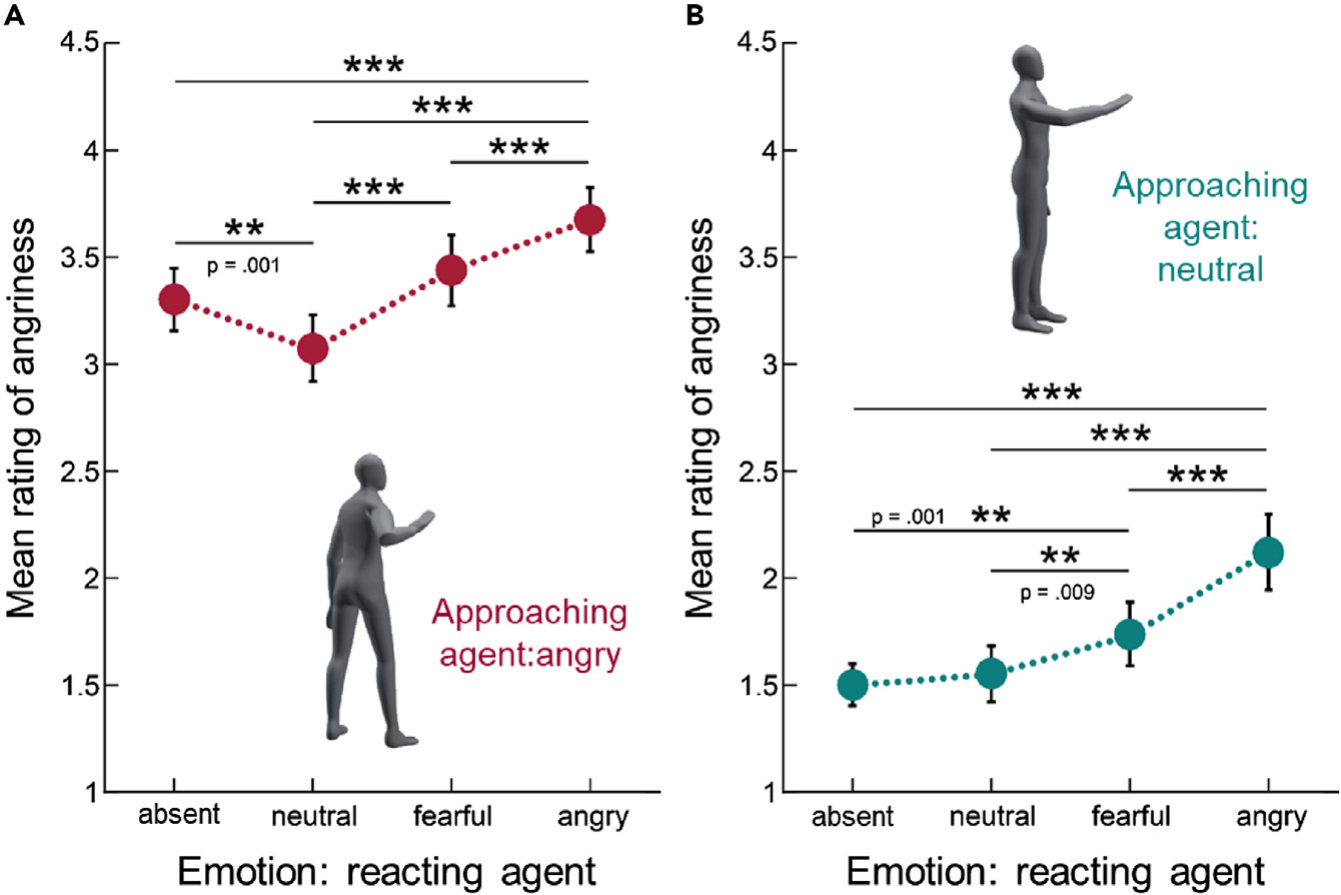
Mean ratings of angriness for the approaching agent A (A) Mean ratings for the angry agent. (B) Mean ratings for the neutral agent. Error bars indicate standard errors (SE). Asterisks mark significant pairwise differences (∗∗p < 0.01, ∗∗∗p < 0.001 Bonferroni corrected). Insets illustrate the general appearance of the agents in the stimuli.

We found that the perception of emotion from individual agents is systematically modulated by the emotion expressed by the interaction partner in a way that reflects meaningfully the social interaction context. Regardless of the emotion of the reacting agent, the angry agents were rated as angrier than neutral agents (Figure 2 effect *Agent-Emotion* (F(1,21) = 299.967, p < 0.001, partial ŋ^2^ = 0.935). Remarkably, the emotion of the reacting actor altered the perception of the acting agent significantly (effect *Reaction* F(3,63) = 54.798, p < 0.001, partial ŋ^2^ = 0.723). Combinations with angry reactions resulted in the highest angriness ratings. Post hoc pairwise comparisons revealed that all acting agents were seen as significantly angrier, if the reaction was angry as well, compared to all other reactions (all p < 0.0001, Bonferroni corrected, Figure 2). Fearful reactions enhanced the perceived angriness of the approaching avatar (angry agent: fearful vs. neutral reaction p < 0.0001; neutral agent: fearful vs. neutral reaction p = 0.009; fearful vs. no reaction p = 0.001, Bonferroni corrected). Most interestingly, we found a significant interaction effect between *Agent-Emotion* and *Reaction* (F(3,63) = 6.574, p = 0.001, partial ŋ^2^ = 0.238). The anger ratings for neutral approaching agents without interaction and in the presence of a neutral reacting agent were not significantly different (p = 1; Figure 2B). By contrast, the anger ratings for angry approaching agents in the presence of neutral reactions were significantly lower compared to all other reactions, including no interaction (p < 0.001; Figure 2A). Here, the neutral reaction seemed to have a soothing effect on the perception of aggressive behavior.

## Discussion

Summarizing, we observed a consistent effect of the second agent’s emotion on the perceived expressiveness of the emotion of the first. This modulation was consistent with the social context of the interaction. Angry as well as fearful reactions increase perceived anger, while neutral reactions substantially diminish anger perceived in the first agent in the sense of de-escalation. These effects are present in visual perception even though the visual features of the individual agents were exactly controlled, and participants had to ignore the second agent. A control experiment (see supplemental information) shows that it is necessary that both agents interact for this modulation to occur.

These results imply that bodily emotions of interacting individuals are visually recognized not simply by an analysis of the visual features of individual agents, even when participants are instructed to ignore other agents in the scene. Instead, interactive emotional expressions of multiple persons seem to be jointly encoded. While it has been shown before those dyads of bodies seem to be visually and neutrally represented differently from individuals when they are interacting,^5^^,^^10^ this is the first study to investigate systematically how visually perceived emotions are influencing each other within interacting dyads.

Our results highlight the importance of taking into consideration genuine interaction factors at play on social agent perception. The understanding of the specific mechanisms of the perception of emotional interactions is also critical for translational work, e.g., on autism spectrum condition,^11^ or for devising efficiently socially interacting robotic systems.

### Limitations of the study

One limitation of our study is that we tested only the effects on the perception of neutral and angry expressions, as fearful approaching actions were not sufficiently reliably rated. Future work will have to investigate if the observed results extend to other classes of emotional stimuli, and in particular to the perception of fearful actions. Also, other types of interactions will have to be explored. In addition, this study investigated the interaction between the emotion expressions in agents that were presented sequentially in time. Similar studies could be devised to investigate how the emotion perception of individual agents is changing if other agents are presented *simultaneously*, while varying their emotional styles. Likewise, one could ask how the emotional style of the approaching sequence modulates bodily emotion perception for the subsequent turning actions. These aspects will have to be topics of future experiments.

## STAR★Methods

### Key resources table

**Table:** 

REAGENT or RESOURCE	SOURCE	IDENTIFIER
**Software and algorithms**		

Motion Builder	Autodesk	https://www.autodesk.de/education/edu-software/overview?sorting=featured&filters=individual
3D Studio MAX	Autodesk	https://www.autodesk.de/education/edu-software/overview?sorting=featured&filters=individual
Nexus	Vicon	https://www.vicon.com/
MATLAB	MathWorks	https://de.mathworks.com

**Other**		

Motion capture data (Motion Builder), recombined stimuli (video clips)	This paper	https://data.mendeley.com/datasets/5y3fgf4j9r/3; https://doi.org/10.17632/5y3fgf4j9r.3

### Resource availability

#### Lead contact

Further information and requests for resources should be directed to and will be fulfilled by the Lead Contact, Martin Giese (martin.giese@uni-tuebingen.de).

#### Materials availability

This study did not generate reagents.

#### Data and code availability

• The datasets from the current study have been deposited in a public repository. Database: https://data.mendeley.com/datasets/5y3fgf4j9r/3.
• Any additional information required to reanalyze the data reported in this paper is available from the lead contact upon request.
• The stimuli datasets have been deposited at Mandalay and are publicly available as of the date of publication. The DOI is listed in the key resources table.

### Experimental model and study participant details

#### Participants

In total, 22 subjects (five males, mean age 24.7 years) participated in the main experiment. In the control experiment reported in the Supplemental Material 12 subjects (4 males, mean age 25.08 years) took part. Participants were recruited for payment, and each of them gave informed consent before participation in the experiment. Participants were informed that their participation was completely voluntary. The study was in accordance with the declaration of Helsinki and approved by the ethics committee of the Eberhard-Karls-University. All data was processed in an anonymous manner.

### Method details

#### Motion capture and computer animation

Animated stimuli were based on motion capture data recorded with a VICON MX system using eight cameras at 120 Hz from two-person emotional interactions. Each actor was wearing 41 reflecting markers. Recorded trajectories were processed and retargeted on a self-made unisex avatar model using the commercial software Autodesk Motion Builder. Animations were created and edited in the 3D animation software Autodesk Maya. We rendered movies with these stimuli with a frame rate of 30fps. The original stimuli and the raw data are publicly available through the data basis [to be added to final version].

### Quantification and statistical analysis

#### A priori sample size estimation

We estimated the minimal sample size for each factor *Agent-Emotion* and *Reaction* individually using the software tool G∗power.^12^ This tool allows for computing the required sample size for repeated measures ANOVA with within subject effects depending on a given probability error *α*, a given *power*, the number of measurements, the number of groups, the correlation between measures, and the desired effect size *f*.

The error probability *α* was set to be 5% and the minimal *power* to be 80%. The number of groups was due to the within-subject design equal to one. Since we used stimuli with two different *Agent-Emotions* and four different possible *Reactions* the values for the number of measurements were 2, respectively 4. The value for the correlation between measures was set to 0.5.

For effect size, we choose a general medium value of 0.25. Since we aimed at studying two different effects, the effect of the *Agent-Emotion*, the effect of the *Reaction,* as well as possible interaction effects, we corrected the effect size for each parameter by the square root of the number of measurements of the second parameter. Thus the desired effect size for *Agent-Emotion* was set to f_Agent-Emotion_ = 0.25∗√4 and the effect size for the *Reaction* to f_Reaction_ = 0.25∗√2.

G∗power estimated the minimal sample size to be 10 for the effect of *Agent-Emotion,* and 13 for the effect of the *Reaction*.

#### Data sharing plan

The datasets from the current study have been deposited in a public repository. Database: https://data.mendeley.com/datasets/5y3fgf4j9r/3.
