# Supplementary material for: Perceptual encoding of emotions in interactive bodily expressions
Source: iScience. 2023 Nov 28;27(1):108548. doi: 10.1016/j.isci.2023.108548 (PMC10755352; doi:10.1016/j.isci.2023.108548)
Supplement: Document S1. Figures S1–S3 [file mmc1.pdf]

iScience, Volume *27*

Supplemental information

Perceptual encoding of emotions

in interactive bodily expressions

Andrea Christensen, Nick Taubert, Elisabeth M.J. Huis in 't Veld, Beatrice de Gelder, and Martin A. Giese

Supplementary Information for: Perceptual encoding of emotional bodily expressions in social interactions

A. Christensen[1], N. Taubert[1], E.M.J. Huis in't Veld[2], B. de Gelder[3], M.A. Giese[1]

[1]Section Computational Sensomotorics, Hertie-Institute for Clinical Brain Research, Centre for Integrative Neuroscience, University Clinic Tübingen, Germany

[2] Department of Medical and Clinical Psychology, School of Social and Behavioral Sciences, Tilburg University, Tilburg, The Netherlands

[3] Brain and Emotion Laboratory, Department of Cognitive Neuroscience, Faculty of Psychology and Neuroscience, Maastricht University, Oxfordlaan 55, EV Maastricht 6229, The Netherlands

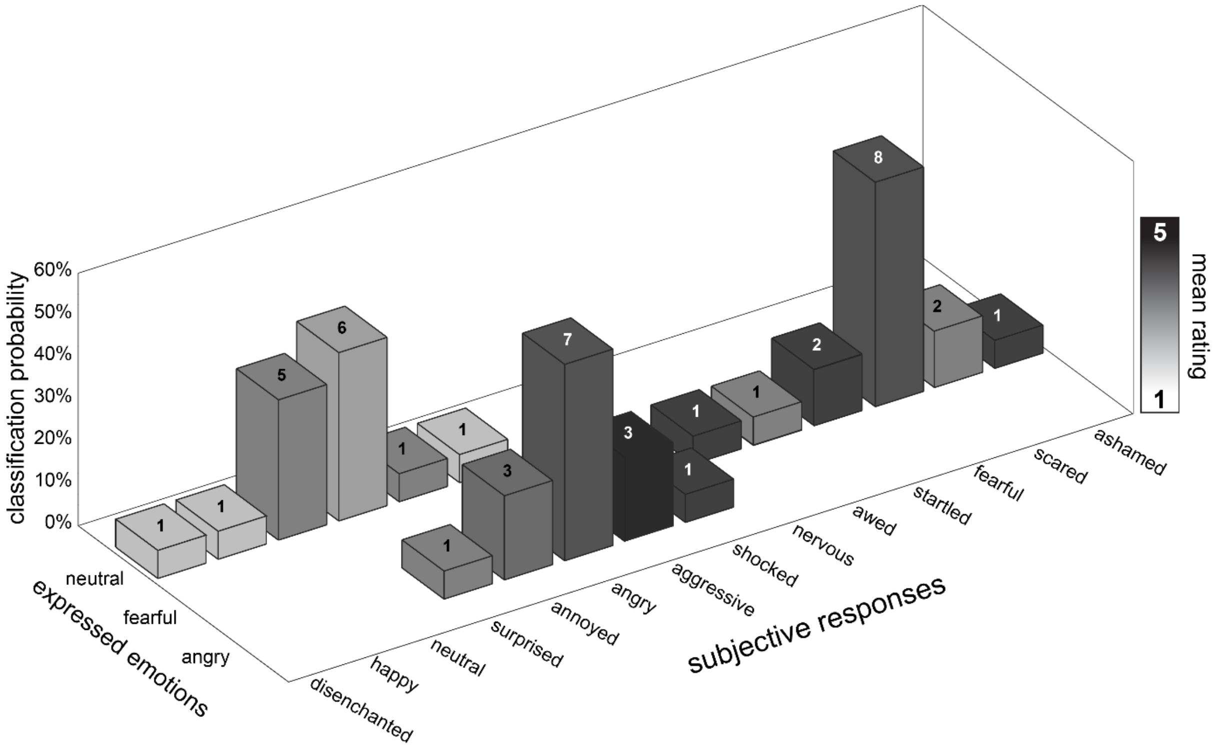

Suppl. Figure 1: Classification and rating results for the three different reacting agents who turned around. In order to verify that the generated animations of the three emotional reactions were perceived according to the expressed emotion we asked 15 new subjects to freely judge the emotion of the three different reactions and to rate their emotion intensity on a scale from 1 to 5. Notably, video clips only showed the reaction, without any initial action. Subjects could observe each stimulus as often as they wanted. No possible answers were given a priori. Instead, we encouraged subjects to report the first emotion that came to their mind. The x-axis indicates the intended emotion of the actors and the y-axis displays the perceived emotion. The height of each bar (z-axis) shows the proportion of answers additionally the numbers in each bar represent the count of responses. Intensity ratings are color-coded: darker colors represent higher mean ratings. Related to Figure 1.

Control Experiment

In order to ensure that the observed effects are driven by the perception of clear interaction of both avatars and not simply by the presence of another agent in the observed scene we conducted a control experiment.

We chose 8 out of the 12 approaching and tapping actions from the first experiment (four angry, four neutral) and the same three reactions (angry, fearful, and neutral). We combined each approaching and tapping action with each emotional reaction (8x3 stimuli), but this time the spatial and temporal relationship between both actions was destroyed by placing the reacting avatar at the opposite side of the stimulus and by starting his reaction 250 ms before the tapping action was finished.

In a first experiment we verified that the applied manipulation of the stimuli really destroyed the perception of an interaction between the two avatars in the scene. We asked 12 subjects (4 males, mean age 25.08 years) to judge the degree of interaction between both avatars in these novel stimuli and the interaction of the two avatars in the corresponding original stimuli used in the main experiment. Subjects rated the degree of interaction on a Likert-Scale ranging from 1 "both actions were completely unrelated" to 5 "both avatars were highly interacting with each other, one action was the consequence of the other". All stimuli were presented 2 times in randomized order, each lasting for 3-5 seconds.

Responses were analyzed with a repeated measures ANOVA using the factors *Agent-Emotion* (angry, neutral), *Reaction* (neutral, fearful, angry) and *Stimulus-Type* (original, de-coupled). We found a highly significant effect of the *Stimulus-Type* (see Suppl. Figure 2; $F(1,11)=56.723$, $p<.001$, partial $\eta^2=.838$). Only the avatars from the original stimuli were perceived as interacting. Further, we observed an interaction effect between the factors *Stimulus-Type* and *Reaction* ($F(2,22)=9.761$, $p=.001$, partial $\eta^2=.470$). For the novel stimuli the interaction ratings were lowest in case of an angry reaction and highest for neutral reactions, whilst for the original stimuli interaction ratings were independent from the reaction. Thus, our novel de-coupled stimuli were clearly perceived as much less interactive than the original stimuli.

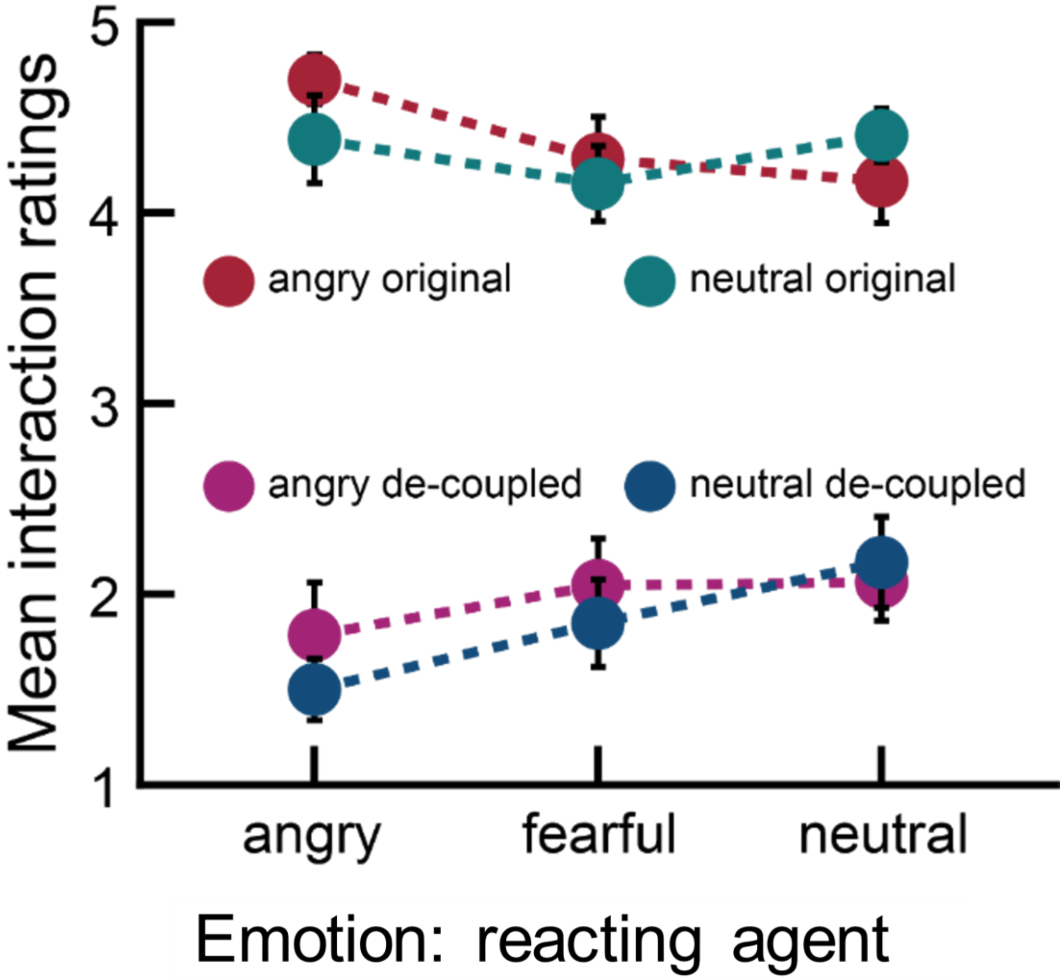

Suppl. Figure 2: Mean ratings of the interaction between both agents in the scene for the original and de-coupled stimuli. Errorbars indicate standard errors (SE). Related to Figure 2.

To test whether the mere presence of the second avatar in the scene - without a clear interaction between both agents - influences the perceived emotion of the first one we conducted another rating experiment. Additionally, to the newly created stimuli, we also used those animations showing only the walking avatar without presentation of a second reacting agent (8 stimuli). All stimuli were presented 3 times in randomized order, each lasting for 3-5 seconds.

Thirteen subjects (five males, mean age 25.2 years) rated the anger expressed by the approaching agent on a Likert-Scale ranging from 1 “not angry” to 5 “extraordinarily angry” by pressing a corresponding key. The number of tested subjects was matching the a priori estimated minimal sample size. We instructed subjects to focus entirely on the walking character and to ignore the others’ reaction. Responses were analyzed with a repeated measures ANOVA using the factors *Agent-Emotion* (angry, neutral) and *Reaction* (none, neutral, fearful, angry).

As expected we found a clear effect of the *Agent-Emotion* on the ratings of anger expressed by the walking avatar (Suppl. Figure 3; effect *Agent-Emotion* ($F(1,12)=168.51$, $p<.001$, partial $\eta^2=.934$). Unlike in the first experiment the reaction of the second avatar did not influence the perception of anger from the walking avatar significantly (effect *Reaction* $F(3,36)=2.067$, $p=.122$, partial $\eta^2=.147$).

These results indicate that it is not the mere presence of the second avatar in the scene that alters the judgement as found in our original experiment, but that both characters need to be perceived as interacting with each other.

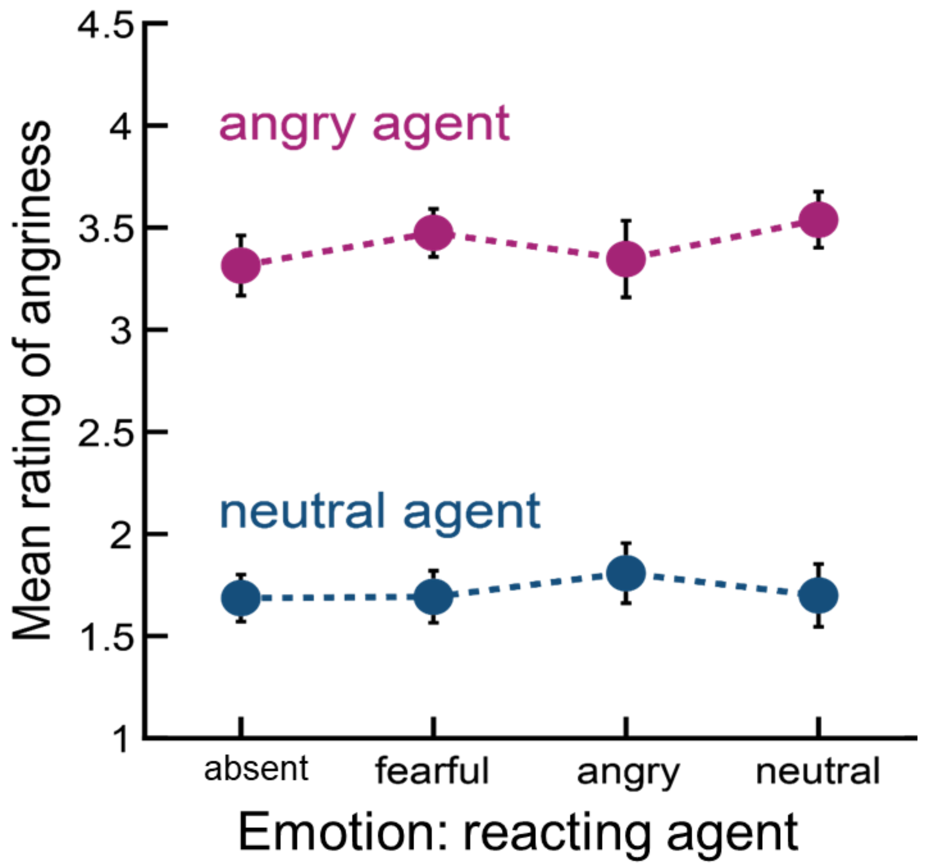

Suppl. Figure 3: Mean ratings of anger for the approaching avatar in presence of another non-interacting agent (showing different emotions), or without a second agent (absent). Error bars indicate standard errors (SE). Related to Figure 2.
